# Mitochondrial respiratory chain deficiency is associated with an impaired skeletal muscle regenerative response and fibrosis in older men with HIV

**DOI:** 10.1038/s41514-025-00273-6

**Published:** 2025-09-23

**Authors:** Matthew Hunt, Amy E. Vincent, Megan M. McNiff, Gareth Ettridge, Caroline Sabin, Alan Winston, Brendan AI Payne

**Affiliations:** 1 Dermatology and Venereology Division, Department of Medicine (Solna), Karolinska Institutet, Stockholm, Sweden; 2 Wellcome Centre for Mitochondrial Research, Newcastle University, Newcastle upon Tyne, UK; 3 John Walton Muscular Dystrophy Research Centre, Translational and Clinical Research Institute, Newcastle Upon Tyne, UK; 4 Centre for Clinical Research, Epidemiology, Modelling and Evaluation (CREME), Institute for Global Health, UCL, London, UK; 5 Department of Genitourinary Medicine & HIV, Imperial Collage Healthcare NHS Trust, St Marys Hospital, London, UK; 6 Section of Virology, Department of Infectious Disease, Imperial College London, London, UK; 7 Department of Infection and Tropical Medicine, Newcastle-upon-Tyne Hospitals NHS Foundation Trust, Royal Victoria Infirmary, Newcastle upon Tyne, UK

**Keywords:** Infectious diseases, Inflammation

## Abstract

Despite suppressive anti-retroviral therapy (ART), some older people with HIV show adverse ageing phenotypes, and the underlying mechanisms remain incompletely understood. We recruited 30 men with HIV aged ≥ 50 years and 15 well-matched men without HIV and performed histological analyses on skeletal muscle biopsies and plasma biomarker measurement, in combination with clinical and functional assessments. Men with HIV showed higher frequencies of frailty, pre-frailty, sarcopenia, and pre-sarcopenia when compared to men without HIV. When assessing skeletal muscle, men with HIV had decreased mitochondrial complex I and IV protein abundance and myofibre regeneration, whilst fibrosis was increased, and plasma TNFα and MCP-4 levels were elevated. Spearman correlation analyses suggested that inflammation and mitochondrial respiratory chain deficiency may result in a damage response in skeletal muscle with resolution by fibrosis rather than regeneration. These findings thus provide plausible rationales for adverse ageing phenotypes in older men with HIV, including frailty and sarcopenia.

## Introduction

Maintenance of skeletal muscle mass and function is of critical importance in healthy human ageing^1^. Sarcopenia is an ageing syndrome characterised by loss of muscle mass and strength^2^, and likewise, the frailty phenotype comprises multiple elements that are at least in part mediated by muscle function^3^. Both sarcopenia and frailty predict adverse health outcomes in the general population, including falls, fractures, hospitalisation, disability, and death^2–4^.

Several pathophysiological processes occur in skeletal muscle during human ageing, which may mediate the age-associated loss of muscle quantity and quality^2^. Perhaps the best described of these is mitochondrial dysfunction^5^. Molecular genetic studies have demonstrated the accumulation of somatic mitochondrial DNA (mtDNA) deletions in skeletal muscle with age^6^, while at the cellular level, individual myofibres from aged individuals show mitochondrial respiratory chain (RC) deficiency^7^. At the functional level, magnetic resonance spectroscopy studies have demonstrated declines in the rate of mitochondrial adenosine triphosphate (ATP) production with ageing^7^. Importantly, these abnormalities in mitochondrial function have been shown to associate with pre-frailty^8^, suggesting that they may lie on the causal pathway leading to the full frailty phenotype^9^. Furthermore, whilst causality is challenging to demonstrate in human ageing studies, mouse models suggest that mitochondrial defects may mediate an accelerated ageing phenotype^6^.

Other pathophysiological processes described in ageing skeletal muscle include fibrosis, increased myofibre degeneration, and decreased regeneration^10^. These cellular processes are part of the damage response within skeletal muscle and are dependent on the limited muscle stem cell (satellite cell (SC)) population^11^.

The population of people with HIV is becoming progressively older, and in most high-income settings, at least 50% of people with HIV are aged 50 years or older^12^. Promotion of healthy or successful ageing is thus of great importance in people with HIV, particularly in light of the fact that people with HIV have an excess of frailty and sarcopenia compared with the general population^13,14^.

Published studies on potential mediators of frailty in people with HIV have primarily examined the role of chronic low-grade inflammation and immune activation^15,16^. In contrast, there is a lack of data on the potential role of skeletal muscle function in adverse ageing phenotypes in people with HIV. The only aspect of skeletal muscle function that has been relatively well studied in the context of HIV is the link between anti-retroviral therapy (ART) and mitochondrial defects^17–19^. Many of the historical nucleoside analogue reverse transcriptase inhibitors (NRTIs), such as zidovudine, stavudine, didanosine, and zalcitabine, inhibit the replication of mtDNA, leading to clinical mitochondrial toxicities such as myopathy, neuropathy, and lactic acidosis^17,20,21^. The role of modern NRTIs, of other ART classes, and of HIV itself in causing mitochondrial dysfunction is not clearly established^22–24^. Many older people with HIV in high-income settings have lived with HIV and received ART for decades^25^, however it is unclear whether people with HIV on suppressive contemporary ART show an excess of mitochondrial defects, if the defects of mitochondrial function or other age-related pathophysiological changes are present in the skeletal muscle of older men with HIV and, if so, whether this may drive frailty and sarcopenia. We therefore examined the role of mitochondrial and other pathophysiological changes in skeletal muscle in both men with and without HIV, before investigating whether these changes may act as a potential mediator of ageing phenotypes in older men with HIV.

Through assessments of clinical, physiological, and demographic data, as well as examinations of both various skeletal muscle pathophysiological features and circulating plasma cytokine levels, our results revealed the putative existence of an abnormal damage response in older men with HIV compared to men without HIV. Our findings that skeletal muscle damage is more readily resolved through fibrosis rather than regeneration in older men with HIV, linked to impaired mitochondrial function, provide a possible rationale for poorer skeletal muscle quality and subsequent excesses of age-related pathophysiological phenotypes such as frailty and sarcopenia in older men with HIV.

## Results

### Cohort clinical characteristics

**Fig. 1:**
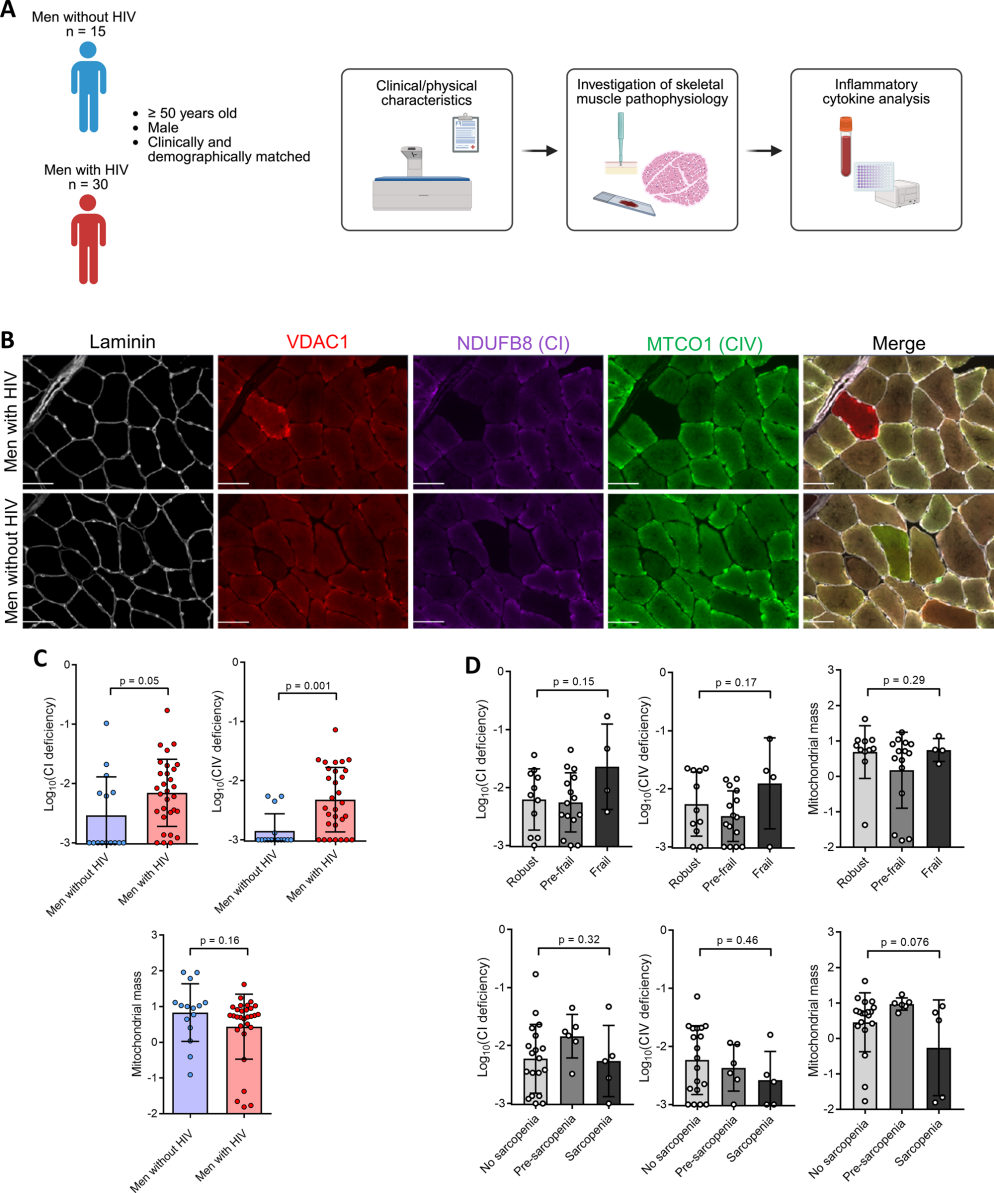
Skeletal muscle mitochondrial RC deficiency and fibre type. **A** Schematic diagram of the workflow. **B** Representative fluorescence image of multiplex immunofluorescence staining of laminin, mitochondrial outer membrane (VDAC1), complex I (CI) (NDUFB8), and complex IV (CIV) (MTCO1) in skeletal muscle. Scale bar = 50 µm. **C** Graphs (mean ± SD) showing a significantly higher proportion of skeletal muscle fibres with CI (*p* = 0.049) and CIV (*p* = 0.001) deficiency in men with HIV (*n* = 30) compared to men without HIV (*n* = 15), as well as mitochondrial outer membrane protein VDAC1 abundance (mean VDAC1 *z*-score). Each dot represents an individual participant. **D** Graphs (mean ± SD) showing proportional CI and CIV deficiency, as well as mitochondrial outer membrane protein VDAC1 abundance in robust (*n* = 11), pre-frail (*n* = 15), and frail (*n* = 4), as well as non-sarcopenic (*n* = 18), pre-sarcopenic (*n* = 6), and sarcopenic (*n* = 5) men with HIV.

We recruited a total of 30 men with HIV, as well as 15 men without HIV who were well-matched for age, lifestyle, and clinical characteristics (Fig. 1A). Clinical and demographic information is detailed in Table 1. Briefly, the median age of men with HIV was 58 years, compared to 59 in men without HIV, whilst men with HIV had controlled CD4 counts (median = 637 copies/μl), a mean of 204 months since diagnosis, 118 months on ART, and 85 months untreated. Thirty per cent were being treated with an ART regimen including a PI, 37% with an NNRTI, 23% with an INSTI, and 37% had previously been treated with known mitochondrially-toxic NRTIs (AZT, ddI, d4T, or ddC).

**Table 1:** Cohort demographic and clinical HIV characteristics

Characteristic	Men without HIV	Men with HIV	*p*
No. of cases	15	30	-
Age (years), median (range)	59 (50–70)	58 (50–85)	0.99
Ethnicity (white) (%)	13 (87)	30 (100)	0.11
CD4 count (cells/µl), median (range)	-	637 (247–1118)	-
Nadir CD4 count (cells/µl), mean (SD)	-	72 (86)	-
Previous AZT, ddI, d4T, ddC exposure, *n* (%)	-	11 (37)	-
Current PI exposure, *n* (%)	-	9 (30)	-
Current NNRTI exposure, *n* (%)	-	11 (37)	-
Current INSTI exposure, *n* (%)	-	7 (23)	-
Months since diagnosis, mean (SD)	-	204 (102)	-
Months on ART, mean (SD)	-	118 (67)	-
Months untreated, mean (SD)	-	85 (89)	-
Alcohol consumption, *n* (%)			0.11
Current	13 (87)	16 (53)	
Former	1 (6.5)	6 (20)	
Never	1 (6.5)	8 (27)	
Alcohol consumption (units), median (range)	9.5 (0–47)	9 (0–60)	0.13
Tobacco use, *n* (%):			0.15
Current	2 (13)	11 (37)	
Former	5 (33)	11 (37)	
Never	8 (53)	8 (27)	
Recreational drug use (current), *n* (%)			0.57
Cannabis	1 (7)	4 (13)	
Other	1 (7)	2 (7)	
HBV or HCV infection, *n* (%)	2 (13)	5 (17)	1
Diabetes mellitus, *n* (%)	3 (20)	3 (10)	0.31
Chronic kidney disease, *n* (%)	0 (0)	3 (10)	0.54
Number of comorbidities, median (range)	1 (0–3)	1 (0–5)	0.34
Number of medications, median (range)	2 (0–6)	3.5 (0–12)	0.09
Polypharmacy, *n* (%)	7 (47)	18 (60)	0.52

Nominal data is expressed as the number and percentage. Ordinal data expressed as the median ± range if not normally distributed or mean ± standard deviation (SD) if normally distributed. *p* values were determined by Fisher’s exact test for nominal data, and either the Mann–Whitney test for non-normally distributed data or the unpaired *t*-test or one-way ANOVA for normally distributed ordinal data.

*AZT* zidovudine, *ddI* didanosine, *d4T* stavudine; zalcitabine; *PI* protease inhibitor, *NNRTI* non-nucleoside reverse transcriptase inhibitor, *INSTI* integrase strand transfer inhibitor, *ART* anti-retroviral therapy, *HBV* hepatitis B virus, *HCV* hepatitis C virus.

In order to evaluate the age-related and skeletal muscle-related function of the participants, we undertook assessments of frailty, sarcopenia, physical performance (SPPB), active status (MET), and body composition according to standardised guidelines. Results from these assessments are shown in Table 2. Here, 13% of men with HIV were classified as frail, whilst 50% were pre-frail. 0% of men without HIV were classified as frail, and 47% were pre-frail (*p* = 0.37). The individual components of the FFP were then examined in order to determine whether fulfilment of any particular criteria was driving frailty. Whilst exhaustion (40%, *p* = 1.00) and grip strength (36.7%, *p* = 0.27) were the highest fulfilled criteria in men with HIV, there was no significant difference when compared to the men without HIV cohort.

**Table 2:** Cohort age-related pathophysiological assessment

Characteristic	Men without HIV	Men with HIV	*p*
**Frailty**, ***n*** **(%)**			0.37
Robust	8 (53)	11 (37)	
Pre-frail	7 (47)	15 (50)	
Frail	0 (0)	4 (13)	
**Sarcopenia,** ***n*** **(%)**			**0.43**
No sarcopenia	15 (100)	19 (63)	
Pre-sarcopenia	0 (0)	6 (20)	
Sarcopenia	0 (0)	5 (17)	
**SPPB,** ***n*** **(%)**			0.32
SPPB high	11 (73)	19 (64)	
SPPB intermediate	4 (27)	10 (33)	
SPPB low	0 (0)	1 (3)	
**MET score,** ***n*** **(%)**			0.92
HEPA active	6 (40)	12 (40)	
Minimally active	7 (47)	12 (40)	
Inactive	2 (13)	6 (20)	
Grip strength (kg), mean (SD)	37.6 (6.6)	35.3 (8.9)	0.39
BMI (kg/m^2^), mean (SD)	32.8 (6.3)	27.1 (3.3)	**0.0003**
**Body composition**			
Waist circumference (cm), mean (SD)	107.8 (14.3)	96.8 (9.9)	**0.0043**
Fat mass, % (SD)	34.1 (7.9)	30.3 (7.8)	0.15
Lean mass, % (SD)	65.9 (7.9)	69.7 (7.8)	0.15
**FFP criteria,** ***n*** **(%)**			
Shrinking	1 (6.7)	2 (6.7)	1
Low activity	2 (13.3)	8 (26.7)	0.32
Exhaustion	6 (40)	12 (40)	1
Grip strength	3 (20)	11 (36.7)	0.27
Slowness	0 (0)	0 (0)	1

Nominal data is expressed as the number and percentage. Ordinal data expressed as the mean ± SD. *p* values were determined by Fisher’s exact test for nominal data, and either an unpaired *t*-test or a one-way ANOVA for ordinal data. Bold values are statistically significant (*p* < 0.05).

*BMI* body mass index, *SPPB* short physical performance battery, *MET* metabolic expenditure, *HEPA* health-enhancing physical activity.

17% of men with HIV were defined as sarcopenic, with 20% pre-sarcopenic; in contrast, 0% of the men without HIV were either sarcopenic or pre-sarcopenic (*p* = 0.43). Additionally, 3% of men with HIV compared to 0% of men without HIV were classified as SPPB low, with 33% and 27% classified as SPPB intermediate, and 63% and 73% as SPPB high (*p* = 0.32), respectively. 20% of men with HIV, compared to 13% of men without HIV, were classified as inactive based on MET score, whilst 40% vs 47% were minimally active respectively (*p* = 0.92). Men without HIV had a significantly higher BMI (*p* = 0.0003) and waist circumference (*p* = 0.0043), but did not differ significantly for percentage fat and lean mass (both *p* = 0.15) and grip strength (*p* = 0.39).

To determine the existence of correlation between physical-related results, as well as with HIV-related clinical characteristics, specifically in men with HIV, we next undertook Spearman’s correlation analyses. Here, no consistent associations were seen between physical phenotypes and HIV-associated parameters in the subgroup of men with HIV (Supplementary Tables 1 and 2).

### Skeletal muscle mitochondrial RC deficiency and pathophysiological defects in older men with HIV

As the maintenance of mitochondrial function, including respiratory function, is important in skeletal muscle and declines with age, as well as the fact that various antiretroviral drugs are known to induce mitochondrial toxicity, we next sought to investigate whether mitochondrial defects contributed to the excess frailty and sarcopenia in men with HIV. Here, through multiplex immunofluorescence staining of mitochondrial RC subunits NDUFB8 (complex I (CI)) and MTCO1 (CIV), as well as the MOM protein VDAC1, it was found that men with HIV had a greater proportion of mitochondrial RC-deficient myofibres compared to men without HIV (Fig. 1B, C). This effect reached statistical significance for CIV (*p* = 0.001) and was marginally significant for CI (*p* = 0.05). There was no significant difference in mean myofibre VDAC1 abundance between the two groups (*p* = 0.16) (Fig. 1C).

When stratifying these results based on how men with HIV were characterised for frailty and sarcopenia, we found that men with both HIV and frailty had numerically higher levels of CI and CIV deficiency compared to men with HIV and either pre-frailty or without frailty, but this effect did not reach statistical significance, and was not seen for sarcopenia (Fig. 1D). In addition, neither CI nor CIV deficiency was significantly associated with exposure to any specific ART class (Supplementary Fig. 1). As alcohol is known as a possible confounder of skeletal muscle and mitochondrial function, we then compared the amount of units consumed per week between men with and without HIV, but found no significant difference (*p* = 0.13) (Table 1). Additionally, Spearman correlation analysis determined there to be no significant association between alcohol consumption and either CI or CIV deficiency in either men with or without HIV (Supplementary Fig. 1E, F).

**Fig. 2:**
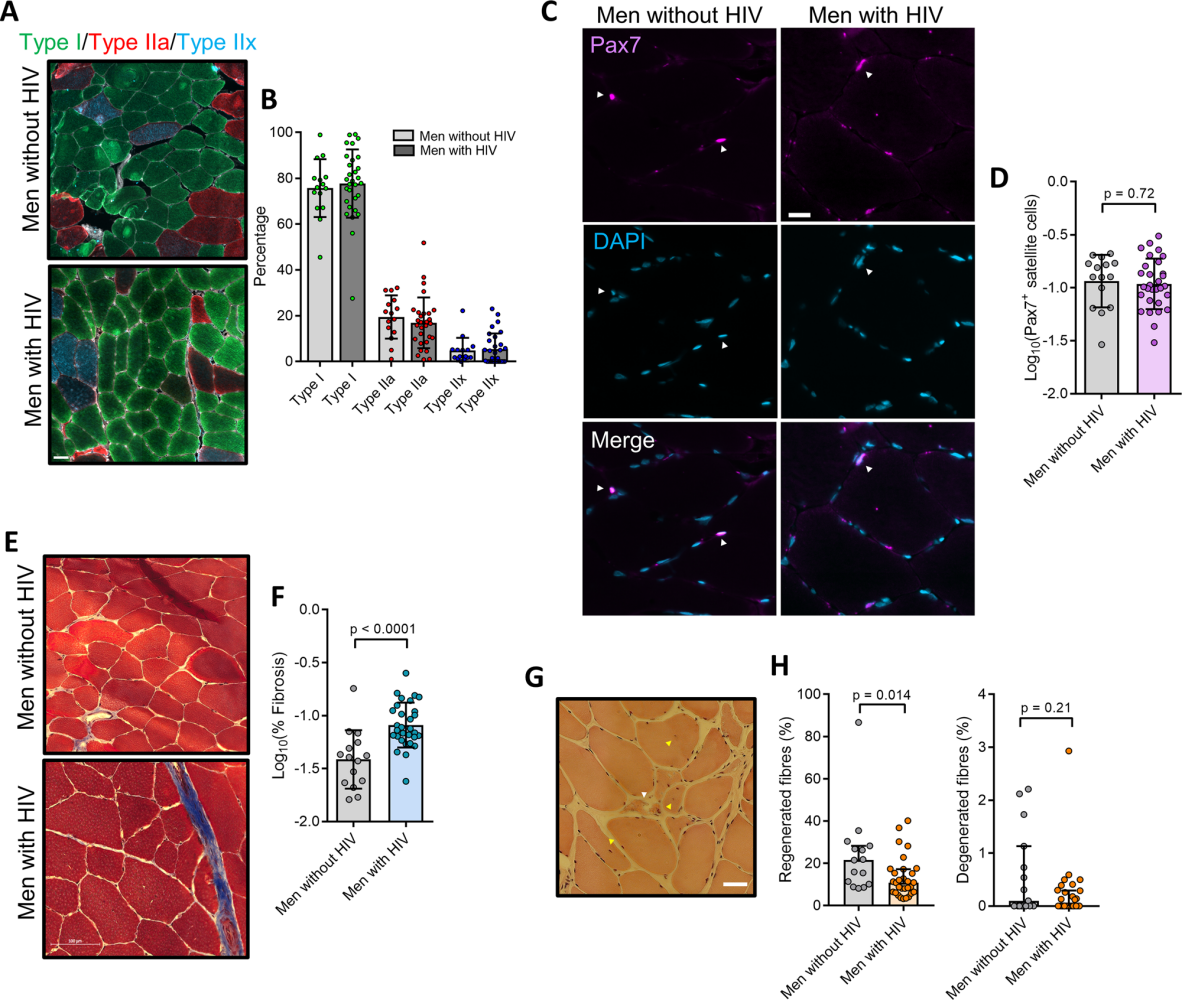
Skeletal muscle pathophysiological assessment. **A** Representative fluorescence images of the three fibre types in men with HIV (n = 30) and men without HIV (*n* = 15). Scale bar = 50 um. **B** Bar graph (mean + SD) showing the percentage of the three respective fibre types in men with HIV and men without HIV. **C** Representative fluorescence images of skeletal muscle cryo-sections stained with Pax7 (SCs) and DAPI (nuclei) in men with (*n* = 30) and without HIV (*n* = 15) individuals. Scale bar = 20 µm. **D** Graph (mean ± SD) showing the frequency of Pax7^+^ SCs per fibre. **E** Representative images of Masson’s trichrome-stained skeletal muscle sections in men with HIV (*n* = 30) and men without HIV (*n* = 15) for the determination of fibrosis. Scale bar = 100 µm. **F** Graph (mean ± SD) showing significantly greater levels of percentage fibrosis in men with HIV compared to men without HIV (*p* < 0.0001). **G** Representative image of a H&E-stained skeletal muscle section showing degenerated (white arrow) and regenerated (yellow arrows demarking myofibres with central nuclei) myofibres. Scale bar = 50 µm. **H** Graph (median ± IQR) of the percentage regenerated fibres in men with HIV (*n* = 30) and men without (*n* = 15), with men with HIV displaying a significantly lower level of regenerated fibres (*p* = 0.02). **G** Graph (median ± IQR) of the percentage degenerated fibres in men with HIV (*n* = 30) and men without HIV (*n* = 15).

Next, in order to better assess the composition of skeletal muscle biopsies, we undertook immunofluorescence staining of the three common fibre types (I, IIa, and IIx). Here, quantification of the proportion of the three common fibre types (Fig. 2A, B) showed no significant difference in the proportion of any fibre type between men with and without HIV (Fig. 2B). In addition, no consistent associations were seen between HIV clinical factors and fibre type composition (Supplementary Table 3).

Following our investigation of fibre type composition, we next sought to investigate whether men with HIV had defects in characteristics associated with dysregulated skeletal muscle regeneration. Here, through immunofluorescence staining of the skeletal muscle Pax7^+^ SCs – a marker for quiescent skeletal muscle stem cells – we found that there was no significant difference in the abundance of Pax7^+^ SCs between men with or without HIV (Fig. 2C, D). However, when assessing collagen deposition through Masson’s Trichome histochemistry, it was found that men with HIV had a significantly higher level of skeletal muscle fibrosis compared to men without HIV (*p* < 0.0001) (Fig. 2E, F). In addition, men with HIV had a lower proportion of regenerated fibres, although this effect was marginally significant when allowing for multiple testing (*p* = 0.014) (Fig. 2G, H), whilst there was no significant difference in the proportion of degenerated muscle fibres between men with and without HIV (Fig. 2H). Finally, we also found that there was no significant difference in the proportion of fibres with IMCL (Supplementary Fig. 2A, B) or in the frequency of lipofuscin granules (Supplementary Fig. 2C, D) between the two groups.

To investigate potential associations between skeletal muscle pathophysiological factors specifically in older men with HIV, we next performed Spearman correlation analyses between the varying factors in men with HIV. Firstly, no associations were found between continuous factors known to be linked to frailty and sarcopenia, such as grip strength, waist circumference, and MET score, with either fibrosis, fibre type composition, Pax7^+^ SCs, or IMCL (Supplementary Table 4). Here, consistent associations were seen between the frequency of Pax7^+^ SCs and the pathophysiological processes observed in skeletal muscle of men with HIV (Supplementary Table 5). Of particular note, higher levels of Pax7^+^ SCs were significantly associated with a decrease in mitochondrial CIV protein abundance (*r* = 0.49, *p* = 0.0056), with greater levels of fibrosis (*r* = 0.53, *p* = 0.002), and with a lower frequency of regenerated myofibres (*r* = 0.54, *p* = 0.002). Overall, whilst these results are based on protein levels of CIV, they suggested the potential existence of a mechanism whereby skeletal muscle regeneration is impaired and subsequently replaced with the formation of fibrotic tissue in men with HIV, potentially underpinned by a decreased abundance of the mitochondrial ETC CIV.

### Plasma biomarkers

**Fig. 3:**
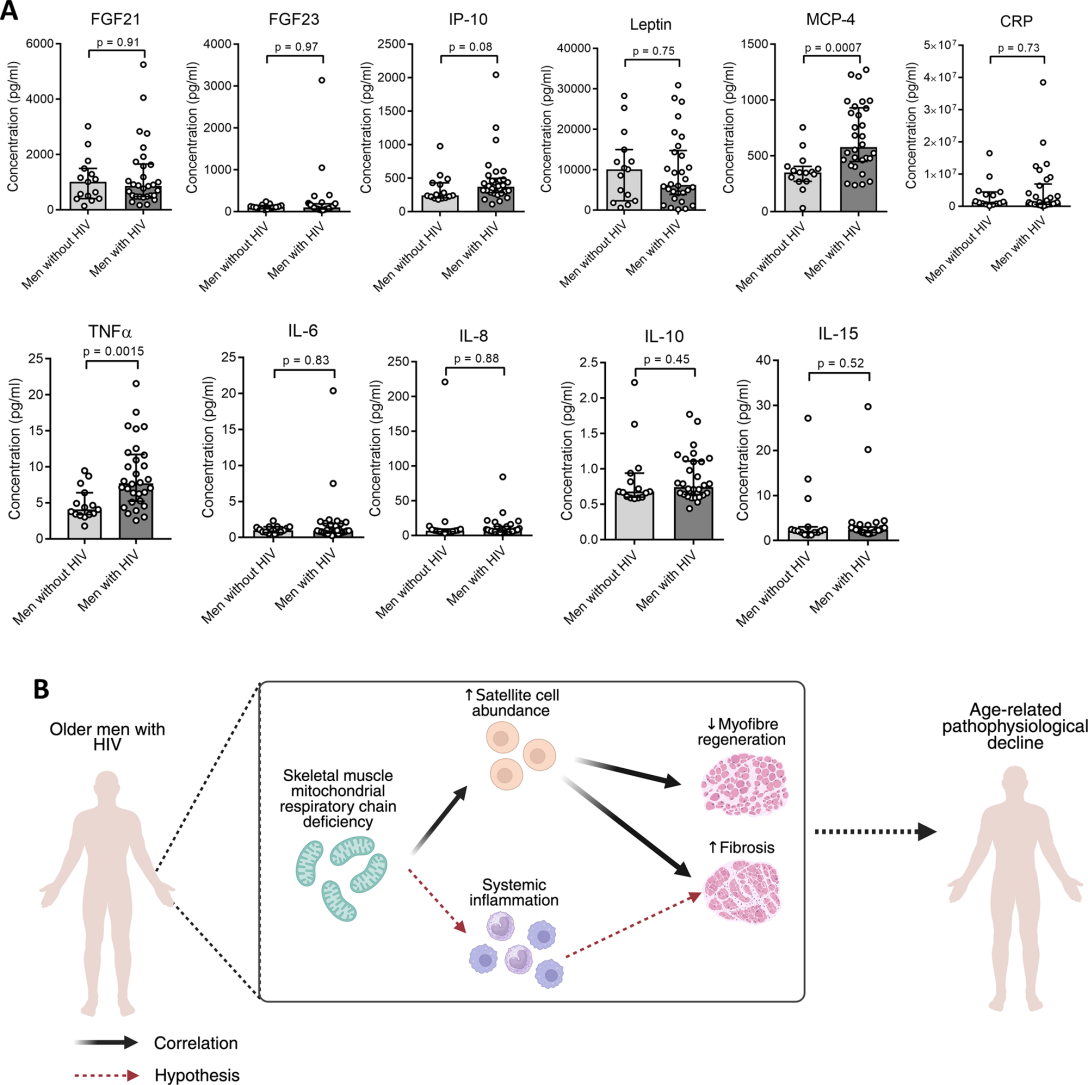
Assessment of inflammatory cytokines and myokines in older men with HIV. **A** Graphs (median ± IQR) showing the concentration (pg/ml) of various inflammatory cytokines, as well as the muscle myokines FGF21 and FGF23 in men with HIV (*n* = 30) and men without HIV (*n* = 15). Each dot represents an individual participant. **B** Schematic summary diagram.

Finally, as chronic but controlled HIV is associated with the increased expression of inflammation markers, we next quantified levels of various plasma biomarkers of systemic inflammation, as well as putative myokines. Here, we found that levels of the proinflammatory cytokines MCP-4 (*p* = 0.0007) and TNFα (*p* = 0.0015) were significantly higher in men with HIV when compared to men without HIV (Fig. 3A). Conversely, levels of the putative myokines, FGF21 and FGF23, did not differ significantly between the two groups (Fig. 3A). When assessing the associations of these increased inflammatory markers with other factors assessed in the study, it was found that MCP-4 or TNFα levels were not correlated with demographic, clinical, or muscle pathophysiological factors amongst men with HIV (Supplementary Tables 6 and 7). However, measures of body fat mass were strongly associated with plasma leptin levels, as expected (Supplementary Table 6).

## Discussion

In this study we attempted to characterise the role of skeletal muscle in adverse ageing phenotypes in older men with HIV. We have combined comprehensive analyses of key pathophysiological processes at the cellular level with functional assessment of frailty and sarcopenia, as well as body composition analysis. Importantly, we carefully included age-, gender-, and lifestyle-matched controls without HIV to minimise potential confounding.

Most participants in our study were aged between 50 and 65 years. In the general population, frailty and sarcopenia are expected to be very uncommon in men of this age^26,27^. More than one-third of men with HIV in our study showed either sarcopenia or pre-sarcopenia, whereas these phenotypes were not seen in any men without HIV. Frailty was uncommon but was seen only in men with HIV. These findings are in keeping with previously published literature on the prevalence of both frailty and sarcopenia in people with HIV^28^. This is despite all men with HIV in our study having well-controlled HIV and good CD4 counts. Most men with HIV had long durations of diagnosed HIV (mean, 17 years), however, long durations of diagnosed but untreated HIV were also common (mean, 7 years).

We identified several pathophysiological processes in the skeletal muscle of older men with HIV. Firstly, we found an excess of myofibres that were deficient in mitochondrial RC abundance. Interestingly, men with HIV with prior exposure to historical NRTIs showed no excess of mitochondrial RC deficiency. This observation is reassuring in that prior exposure to NRTIs, which inhibit the mtDNA polymerase gamma, does not appear to lead to a legacy effect of permanent mitochondrial defects^22^. Conversely, it suggests that other factors are driving mitochondrial defects in older men with HIV on suppressive contemporary ART. This could include a milder chronic effect of contemporary NRTIs or other ART classes over prolonged periods of time. We were not able to detect an effect of any specific class of ART. However, owing to the size of the study and the complex heterogeneity of current and prior ART histories, such an effect cannot be fully excluded. Alternatively, it may be that there are systemic features of treated HIV infection that promote muscle mitochondrial defects. For example, low-grade chronic inflammation is well recognised in people with HIV, as is not fully normalised by suppressive ART^29^. With this in mind, we analysed a panel of plasma biomarkers and observed higher levels of pro-inflammatory cytokines, including TNFα, in men with HIV, as has been reported elsewhere^29^. Future studies should further examine potential links between chronic inflammation and mitochondrial defects in people with HIV, as well as further investigate the link between these factors and inclusion body myositis (IBM) pathologies^30^. Interestingly, recent data have suggested mechanisms by which inflammation might lead to mitochondrial defects in skeletal muscle, mediated via the indoleamine 2,3-dioxygenase 1 (IDO1) pathway^31^. Conversely, the presence of mitochondrial defects might also promote systemic inflammation, as for example, the release of mtDNA into the cytosol is known to lead to inflammasome activation, which in turn increases proinflammatory cytokine production^32,33^.

Secondly, we demonstrated that older men with HIV have increased skeletal muscle fibrosis. Fibrosis is a response to tissue damage common to most tissues, and limited published data have previously reported increased fibrosis in people with HIV^34^. Fibrosis has been shown to result from the resolution of an inflammatory response within a tissue through anti-inflammatory responces^10,35,36^. Although fibrosis was not directly associated with elevated TNFα levels in our study, the observed excess of the proinflammatory cytokine TNFα suggests the potential of an indirect role, and thus may be relevant in the development of fibrosis in people with HIV.

A healthy response to myofibre damage is regeneration, a process which relies on muscle stem cell (SC) function^10^. We thus measured the frequency of degenerated and regenerated myofibers, along with muscle stem cell abundance, and demonstrated that regeneration was decreased in men with HIV compared to men without HIV. This suggests that the normal damage response in skeletal muscle may be impaired in men with HIV, as numerically, stem cell abundance did not differ between men with and without HIV. It is important to note that our assay measured the proportions of degenerated and regenerated myofibres and of stem cells. It did not measure their dynamic response to an injury challenge, as can be performed in rodent or intervention studies^34^. Additionally, whilst the quantification of mitochondrial ETC complexes I (CI) and IV measured the abundance of subunits of these complexes within whole skeletal muscle fibres, it did not measure changes at the individual cellular level. Nevertheless, we observed consistent associations between SC abundance and the pathophysiological abnormalities seen in the skeletal muscle of older men with HIV—decreased mitochondrial RC protein abundance and myofibre regeneration, as well as increased fibrosis. As previous studies have shown that mitochondrial defects lead to abnormal stem cell response to muscle damage^11^, this suggests a plausible unifying mechanistic link between these observations. Mitochondrial dysfunction may explain the observed reduction in myofibre regeneration, despite normal stem cell numbers. Mitochondrial function is important for the determination of SC fate^37^, and TCA (citric acid) cycle intermediates are essential cofactors needed for the epigenetic remodelling required for SC commitment and differentiation^11,38^. Furthermore, poor muscle regeneration has been shown to lead to an increase in fibrotic tissue. This, in turn, is associated with a decline in contractability and muscle strength^10^. Taken together, our observations of increased fibrosis and decreased myofiber regeneration in men with HIV, which correlated with decreased mitochondrial RC protein abundance, suggest an alternative damage response leading to impaired skeletal muscle quality (Fig. 3B).

As an observational study using skeletal muscle biopsies, our analyses are limited by a small sample size. This limited the power to detect significant within-group associations, including the effects of different ART classes, such as d-drugs, and the predictors of frailty and sarcopenia. In addition, although the study population was well-matched for age, gender, and lifestyle, we acknowledge the potential presence of other variables that may not have been captured, such as insulin resistance. As the study group was all men with well-controlled HIV, our results may not be applicable to other settings, and it will be particularly important to include older women with HIV in future research.

Overall, our findings suggest that mitochondrial function is likely to have an important role in mediating pathophysiological factors that underpin skeletal muscle function and thus adverse ageing phenotypes in older men with HIV.

## Methods

### Patient cohort

This study was approved by the local research ethics committee (Newcastle and North Tyneside 2, 17-NE-0015) and performed in accordance with the Declaration of Helsinki. As a separate, new study, skeletal muscle samples were obtained by percutaneous biopsy of tibialis anterior under local anaesthesia from males with (*n* = 30) and without (*n* = 15) HIV. Some were recruited in conjunction with the Pharmacokinetics and Clinical Observations in People over Fifty (POPPY) study^39^. All participants were 50 years or older. In addition, participants supplied 30 ml of whole blood. Men with HIV were able to participate if they had been on ART for at least 6 months and had a suppressed plasma HIV-1 viral load (<200 copies/ml).

All participants completed a standardised interview, and clinical information (including, for those with HIV, CD4+ lymphocyte count, HIV diagnosis details, and ART history) was collected and confirmed through medical records, where available. The presence or absence of comorbidities was confirmed through medical records. Written consent was obtained from all participants.

The presence or absence of the following comorbidities was assessed through patient questionnaire: stroke and CVD, neuropathy, diabetes, dementia, cancer, renal disease, fractures, hepatitis, peripheral vascular disease, joint disease or replacements, osteoporosis, and falls. Clinical HIV data were captured by patient questionnaire and case note review and included current CD4 count, months since diagnosis, months on ART, and months untreated.

### Assessment of ageing phenotype

Frailty was assessed as previously described using the original five Fried’s frailty phenotype (FFP) criteria^3^. Participants with one or two criteria were classified as pre-frail. Physical function was determined through a standardised short physical performance battery (SPPB) consisting of a repeat chair stand, standing balance test, hand grip assessment, and 4 m walk, with scoring described previously in ref. ^40^.

Sarcopenia was classified based on muscle mass (DXA), strength (grip strength), and physical performance (SPPB)^2^. Participants were classified as having pre-sarcopenia if they had abnormal results for muscle mass only; sarcopenia if they had abnormal results for muscle mass, as well as either muscle strength (classified as weak grip strength) or physical performance; and severe sarcopenia if they had abnormal results from all three criteria, as based on EWGSOP2 classifications.

Metabolic equivalent (MET) expenditure per week was calculated as a surrogate for physical activity, with participants classified as either ‘inactive’, ‘minimally active’, or ‘HEPA active’. Results were calculated as previously described in ref. ^41^.

### Skeletal muscle histology and immunofluorescence

Transverse snap-frozen percutaneous biopsy sections (10 µm) were subjected to an array of immunofluorescence assessments in order to analyse mitochondrial RC CI and IV (CIV) deficiency, mitochondrial outer membrane (MOM) protein VDAC1 abundance, intramyocellular lipid accumulation (IMCL), Pax7^+^ SC frequency, fibre type composition, and lipofuscin granule deposition. In addition, histology was performed to determine skeletal muscle fibrosis, as well as myofibre degeneration and regeneration.

Specifically, CI and CIV deficiency were quantified using a multiplex immunofluorescence assay as described previously in ref. ^42^, with markers for laminin (myofibre boundary) (Sigma, #L9393); VDAC1 (mitochondrial outer membrane marker) (Abcam, #ab14734); NDUFB8 (mitochondrial RC complex I) (Abcam, #ab110242); and MTCO1 (mitochondrial RC complex IV) (Abcam, #ab14705). Secondary antibodies included AlexaFluor 405 (Laminin) (Invitrogen, #A31556); AlexaFluor 546 (VDAC1) (Thermo Fisher Scientific, #A21143); Anti-IgG1-Biotin (Jackson Laboratories, #115-065-205) plus Strep-conjugated AlexaFluor 647 (both NDUFB8) (Thermo Fisher Scientific, #S-32357); AlexaFluor 488 (Life Technologies, #A21131). For each batch of skeletal muscle analysed, additional cryosections were stained in parallel using only the primary antibody for laminin, along with all the secondary antibodies (termed ‘no primary controls’, NPC). In order to minimise batch effects, all batches of cryosections analysed comprised a mixture of all experimental groups, NPC, and calibrator samples. For analysis, fibres were segmented using the laminin signal, before the average fluorescence intensity for NDUFB8, MTCO1, and VDAC1 was automatically quantified using in-house software (MATLAB 2015a). NDUFB8 and MTCO1 intensity was then expressed relative to VDAC1 before the abundance of each respective protein was expressed as *z*-scores based on the expression from young healthy calibrator samples using an in-house R Shiny script. Myofibres with NDUFB8 or MTCO1 *z*-scores < −3 were classified as ‘deficient’ and >−3 as ‘normal’. Finally, the percentage of myofibres with either NDUFB8 or MTCO1 deficiency was log_10_ transformed. Fluorescence images for RC complex abundance, as well as all other immunofluorescence imaging, were acquired on a Zeiss Axio Imager M1 at 20× magnification and tiled and analysed on Zen 2011 Blue Edition (Zeiss).

IMCL was determined through BODIPY (493/503) (Thermo Fisher Scientific) staining and imaging. After air-drying at room temperature (RT) and fixation with 3.7% formaldehyde (ChemCruz) for 30 min each respectively, sections were permeabilised with 0.25% Triton x-100 (Thermo Fisher Scientific) for 5 min then incubated with antibody against laminin (Sigma, #L9393) for 60 min at RT before incubation with AlexaFluor 405 (Invitrogen, #A31556) secondary antibody and BODIPY (493/503) (Thermo Scientific Fisher, #D3822) for 90 min in a dark humidified chamber at RT. Finally, sections were mounted in Molwoil 4–88 (Sigma) and stored at −20 °C. For image analysis, individual myofibres were qualitatively classified as having IMCL or being ‘normal’ depending on BODIPY staining coverage and fluorescence intensity.

For the quantification of Pax7^+^ SC, 10 µm cryo-sections were air-dried for 1 h at RT before fixation in cold 4% PFA for 4 min. Next, sections were permeabilised for 10 min at RT with 0.2% Trion-x 100 before blocking with 5% NGS for 1 h at RT, followed by overnight incubation with Pax7 primary antibody (diluted in 10% NGS) (DSHB) at 4 °C. The following day, sections were incubated with AlexaFluor 488 (Thermo Fisher Scientific, #A-21242) secondary antibody for 2 h at RT before incubation with Hoerst (1:1200) (abcam) for 15 min at RT and finally mounting with Prolong Gold Antifade Mountant (Thermo Fisher Scientific). Stained sections were imaged before the frequency of Pax7^+^ SCs per fibre was quantified following confirmation of Pax7 and nuclei colocalisation.

To quantify the proportion of skeletal muscle fibre types I, IIa, and IIx, air-dried cryo-sections were subjected to multiplex immunofluorescence staining with targets for the respective fibre types. After air-drying of cryo-sections for 1 h at RT, sections underwent fixation with 4% paraformaldehyde (PFA) for 1 h at RT before blocking with 10% NGS (Abcam) again for 1 h at RT, followed by incubation with a primary antibody cocktail with markers for laminin (Sigma, #L9393), BA-F8 (type I) (DSHB, #10572253), SC-71 (type IIa) (DSHB, #2147165), and 6H1 (type IIx) (DSHB, #2314830) overnight at 4 °C. Next, sections were incubated with a secondary antibody cocktail consisting of AlexaFluor 405 (laminin) (Invitrogen, #A31556); AlexaFluor 488 (BA-F8) (Invitrogen, #A31141); AlexaFluor 546 (SC-71) (Invitrogen, #A21123); and AlexaFluor 647 (6H1) (Invitrogen, #A21238) for 2 h at 4 °C. Sections were then mounted using ProLong Gold Antifade Mountant (Thermo Fisher Scientific) and stored at −20 °C prior to imaging. Following imaging, the number and proportion of each respective fibre type were quantified based on fluorescence staining profile.

Next, in order to quantify lipofuscin granule accumulation, cryo-sections were air-dried for 1 h and immediately mounted on coverslips. Sections were imaged at 546 nm and 647 nm wavelengths at 20× magnification and tiled before the frequency and area of autofluorescent lipofuscin granules were quantified on the Columbus Image Data Storage and Analysis System software based on colocalisation of both respective channels.

To quantify degeneration and regeneration of muscle fibres, 10 µm cryo-sections were subjected to Haematoxylin and Eosin (H&E) staining, while fibrosis was assessed by Masson’s trichrome staining. With regards to H&E histochemistry, 10 µm cryo-sections were removed from −80 °C and air-dried for 1 h at RT. The following sections underwent fixation with cold 4% PFA for 3 min. Next, sections were stained with Haematoxylin for 10 min, then rinsed clear in H_2_O before being washed in Scott’s tap water for 1 min, followed by Eosin for 1 min. Finally, sections were rehydrated through an EtOH gradient (10 dips 70% EtOH, 10 dips 95% EtOH, 20 dips 100% EtOH) followed by 2 changes of 20 dips in Histoclear, mounting with DPX (Sigma), and storage at RT.

For Masson’s trichrome histochemistry, 10 µm cryo-sections were removed from −80 °C and air-dried for 1 h at RT before fixation as described previously, followed by further fixation in Bouin’s Fluid (Sigma) at 60 °C for 30 min before being rinsed clear with H_2_O. Next, sections were stained with Weigert’s Iron Haematoxylin (Abcam) for 5 min, rinsed clear with H_2_O, then differentiated in phoshotunsic acid solution (Abcam) for 10 min. After rinsing with H_2_O, sections were incubated with alanine blue (Abcam) for 7 min followed by differentiation with acetic acid (Abcam) for 3 min. Finally, sections were rehydrated by an EtOH gradient and mounted as described above.

For imaging, both H&E and Masson’s trichome-stained sections were imaged using a Zeiss Axio Imager M1 and Zen 2011 (blue edition) with a chromatic digital camera (AxioCam MRm) at 10x magnification. Regenerated myofibres were determined by the presence of central nuclei, whilst degenerated myofibres were qualitatively determined as described previously in ref. ^43^. Briefly, this included either loss of cross-striations, cytoplasmic eosinophilia, fibre fragmentation or rupture, or sarcoplasmic vacuolisation. Fibrosis was quantified as the percentage area covered by collagen (blue staining).

### Cytokine and myokine quantification

A bespoke panel of inflammatory cytokines, as well as putative myokines and mitokines, was measured using the MSD U-PLEX (MCP-4, Leptin, TNFα, IL-10, IL-6, IL-8, IL-15, IP-10, and CRP) and V-PLEX (FGF21 and FGF23) assays (Meso Scale Discovery) according to the manufacturer’s instructions, with signal intensity read on the MESO QuickPlex SQ 120 (Meso Scale Discovery).

### Statistical analysis

Statistical analysis was performed in Prism v5.04, IBM SPSS Statistics v23, Columbus Image Data Storage and Analysis System, and Microsoft Excel 2016. Shapiro–Wilk tests were used to determine normality within datasets, with a *p* ≥ 0.05 indicating normality. One-way ANOVA or Kruskal–Wallis test and unpaired *t*-tests or Mann–Whitney test were used to compare differences between groups. Fisher’s exact test was used to determine differences between categorical factors. Spearman’s correlation analysis was performed to compare continuous variables. To account for multiple testing, a Bonferroni correction was performed within families of related assays.

Specifically, proportions between groups (men with and without HIV) were compared by Fisher’s Exact test.

Univariate analysis between continuous variables for clinical HIV data, characteristics related to physical function (including grip strength, waist circumference, fat and lean mass, BMI), and muscle pathophysiology parameters was performed using Spearman’s correlation.

Groupwise comparison of continuous variables (including biomarkers) was performed by the Mann–Whitney test. One-way ANOVA (normally distributed) or Kruskal–Wallis test (non-normally distributed) was performed in order to determine the associations between the categorical measures of physical function (frailty phenotype, sarcopenia, SPPB, and MET) with clinical HIV data.

Bonferroni correction for multiple testing was performed. Bonferroni-corrected *p* values were calculated for families of related analyses, as follows. For correlation analyses of HIV clinical data, a Bonferroni-corrected *p* value < 0.01 was determined to be statistically significant. For correlation analyses of skeletal muscle pathophysiological factors, we applied a corrected *p* value < 0.0062, and for inflammatory biomarkers analyses, a corrected *p* value < 0.0045.

As this was an exploratory study to describe histochemical changes in skeletal muscle, we additionally focused on the mechanistic consistency of observed associations.

## Supplementary information

Supplementary file

## Data Availability

All raw data used in the analysis of patient clinical, demographic, physical function, inflammatory cytokine analysis, and skeletal muscle assessment can be found in the public repository: 10.6084/m9.figshare.29715035.
