# Supplementary material for: Mitochondrial respiratory chain deficiency is associated with an impaired skeletal muscle regenerative response and fibrosis in older men with HIV
Source: NPJ Aging. 2025 Sep 23;11(1):79. doi: 10.1038/s41514-025-00273-6 (PMC12457611; doi:10.1038/s41514-025-00273-6)
Supplement: Supplementary file 1 — Supplementary file [file 41514_2025_273_MOESM1_ESM.pdf]

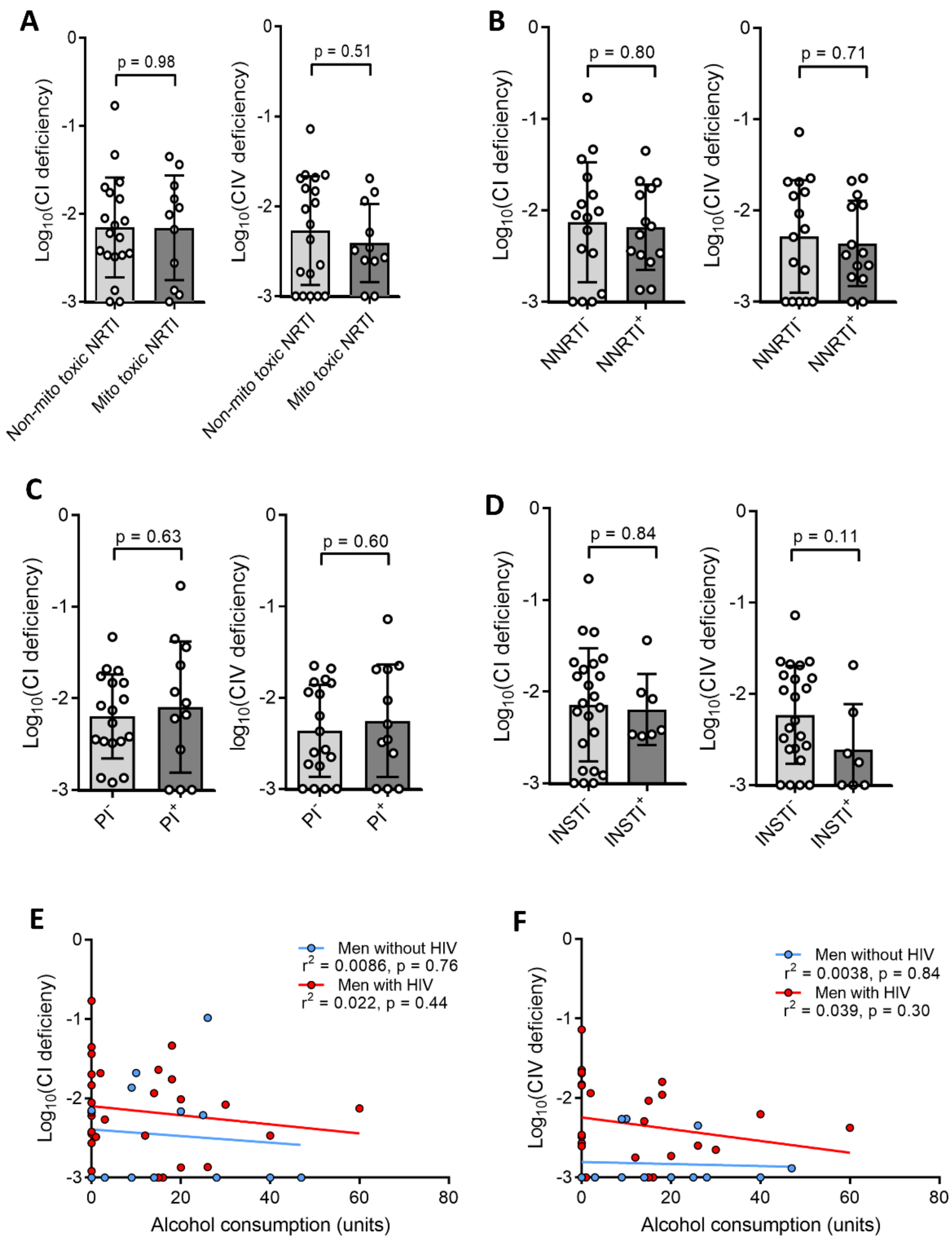

Supplementary Figure 1. Mitochondrial respiratory chain deficiency is not associated with ART class.

Graphs showing no significant difference in either log-transformed myofibre CI or CIV deficiency between groups of men with HIV when delineating them into whether they had been exposed to (**A**) mitochondrially-toxic nucleoside reverse transcriptase inhibitors (NRTIs) (zidovudine, stavudine, didanosine, zalcitabine) or other NRTIs, (**B**) non-nucleoside reverse transcriptase inhibitors (NNRTIs), (**C**) protease inhibitors (PIs), or (**D**) integrase inhibitors (INSTI). Each dot represents an individual.

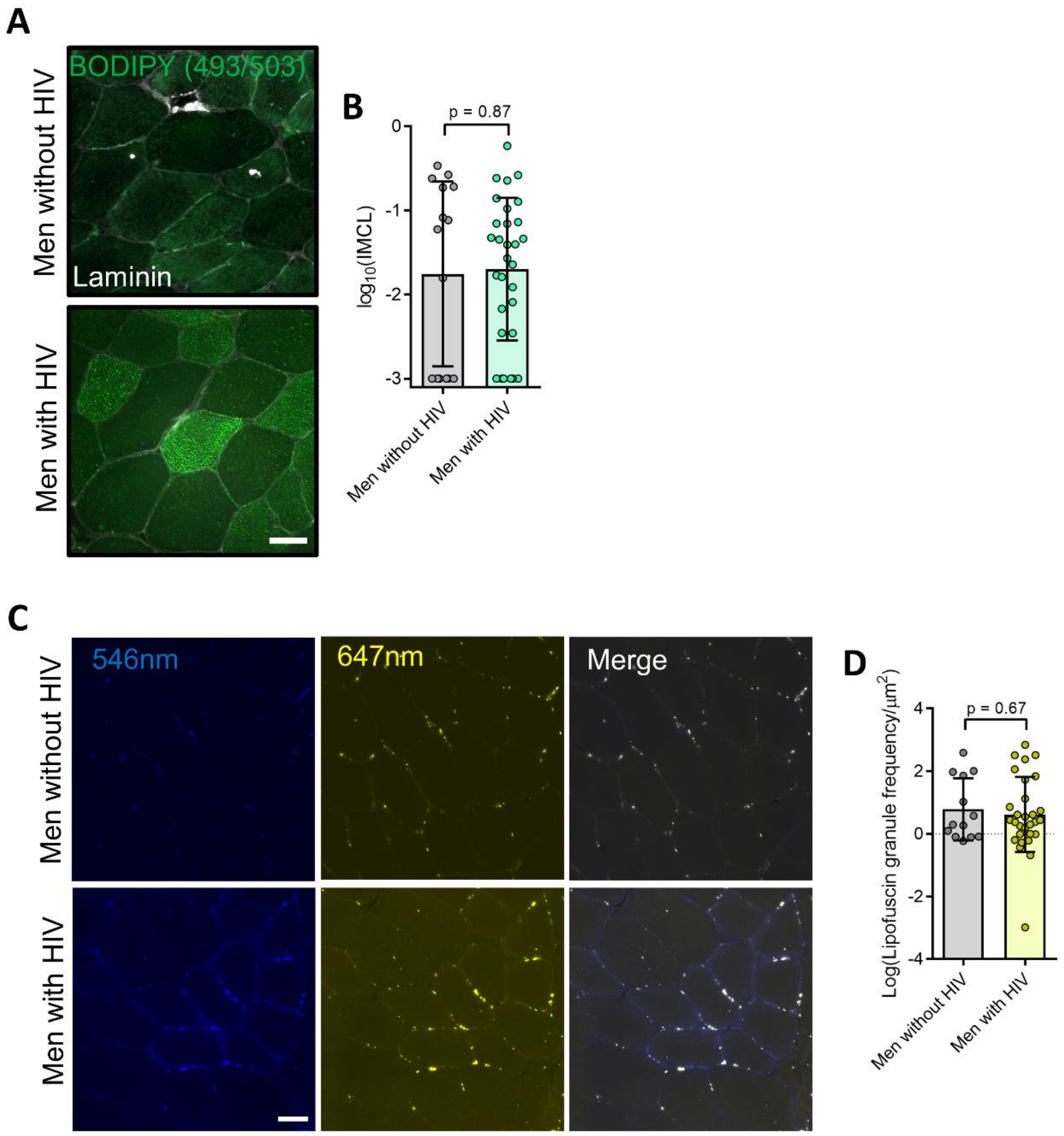

Supplementary Figure 2. Immunofluorescence analysis of skeletal muscle pathophysiological features.

(**A**) Representative fluorescence image of BODIPY (493/503) stained men with (n = 30) and without HIV (n = 15) skeletal muscle sections. Scale bar = 50µm.

(**B**) Graph (median ± IQR) showing the proportion of myofibres with IMCL in men with and without HIV.

(**C**) Representative fluorescence images of lipofuscin granules following detection of autofluorescence at 546 and 647nm wavelengths in men with (n = 29) and without HIV (n = 13). Scale bar = 50µm.

(**D**) Graph (median ± IQR) showing no significant difference in the frequency of lipofuscin granules after normalisation to area (μm^2) in men with and without HIV.

Characteristic	Months since diagnosis		Months on ART		Months untreated		CD4 count (cells/µl)		BMI (kg/m²)		Waist circumference (cm)		Grip strength (kg)		Fat mass (%)		Lean mass (%)	
	r	p	r	p	r	p	r	p	r	p	r	p	r	p	r	p	r	p
Months since diagnosis	-	-	0.52	0.003	0.74	<0.0001	-0.045	0.82	-0.16	0.39	-0.16	0.40	-0.40	0.027	-0.25	0.19	0.25	0.19
Months on ART			-	-	-0.038	0.84	-0.25	0.20	-0.417	0.022	-0.10	0.59	-0.018	0.93	-0.16	0.41	0.16	0.41
Months untreated					-	-	0.06	0.78	0.005	0.98	-0.13	0.49	-0.46	0.011	-0.13	0.51	0.13	0.51
CD4 count (cells/µl)							-	-	0.45	0.02	-0.013	0.95	-0.16	0.43	0.16	0.42	-0.16	0.42
BMI (kg/m²)									-	-	0.69	0.000	-0.11	0.568	0.48	0.007	-0.48	0.007
Waist circumference (cm)											-	-	0.036	0.85	0.59	0.001	-0.59	0.001
Grip strength (kg)													-	-	-0.13	0.49	0.13	0.49
Fat mass (%)															-	-	-1.0	0.00
Lean mass (%)																	-	-

Supplementary Table 1. Associations between continuous clinical and body composition factors in men with HIV.
Spearman's unadjusted univariant correlation analysis with continuous clinical and body composition factors in men with HIV (n = 30).

Characteristic	Frailty				Sarcopenia				SPPB				MET			
	Frail	Pre-frail	Robust	p	Sarcopenia	Pre-sarocpenia	No-sarcopenia	p	Low	Intermediate	High	p	Inactive	Minimally active	HEPA active	p
Months since diagnosis	243 (129)	211 (106)	181 (92.9)	0.57	238 (170)	245 (74.9)	182 (87.4)	0.31	180 (96.2)	244 (111)	266 (0)	0.24	214 (87.2)	167 (92.9)	259 (136)	0.19
Months on ART	93.8 (41.9)	114 (62.5)	133 (81.6)	0.58	103 (53.2)	177 (52.5)	104 (67)	0.052	118 (74.1)	120 (60)	99 (0)	0.96	133 (78.3)	110 (67.8)	106 (44.1)	0.65
Months untreated	149 (126)	103 (81.4)	52.5 (74.2)	0.12	135 (150)	65 (61.8)	87.8 (75.3)	0.38	64.3 (55.5)	137 (124)	167 (0)	0.13	81.3 (63.8)	66.7 (84.9)	153 (122)	0.09
CD4 count (cells/µl)	542 (261)	734 (227)	592 (208)	0.19	656 (156)	671 (218)	650 (263)	0.98	643 (238)	684 (239)	584 (0)	0.87	660 (330)	637 (131)	686 (228)	0.92

Supplementary Table 2. Associations between clinical HIV factors and age-related pathophysiological classifications in men with HIV.
Table depicting One-Way ANOVA analysis of various clinical HIV factors when grouped into respective classifications of either frailty, sarcopenia, SPPB, and MET score groups in men with HIV (n = 30).

Characteristic	Months since diagnosis		Months on ART		Months untreated		CD4 count	
	r	p	r	p	r	p	r	p
Log10(CI deficiency)	0.25	0.19	0.28	0.14	0.17	0.36	-0.27	0.17
Log10(CIV deficiency)	0.21	0.26	0.26	0.17	0.17	0.37	-0.14	0.48
VDAC1 abundance	-0.18	0.33	0.084	0.66	-0.24	0.21	-0.18	0.37
Type I (%)	-0.24	0.20	0.018	0.92	-0.29	0.12	-0.11	0.56
Type IIa (%)	0.14	0.47	-0.043	0.82	0.20	0.29	0.046	0.82
Type IIx (%)	0.40	0.029	0.14	0.46	0.36	0.054	0.19	0.32
Pax7	0.21	0.26	0.17	0.37	0.21	0.28	-0.033	0.87
Fibrosis	0.22	0.26	-0.076	0.69	0.26	0.17	-0.004	0.99
Regeneration	0.31	0.092	0.014	0.94	0.28	0.14	-0.13	0.53
Degeneration	0.081	0.67	0.19	0.31	0.005	0.98	-0.12	0.54
IMCL	0.12	0.53	-0.022	0.91	0.086	0.65	0.28	0.15
Lipofuscin frequency	0.058	0.76	0.17	0.37	0.055	0.78	0.27	0.17

Supplementary Table 3. Associations between HIV factors and muscle mitochondrial analysis in men with HIV.
Spearman’s unadjusted univariant correlation analysis with continuous HIV clinical factors and either CI deficiency, CIV deficiency, or mitochondrial outer membrane protein VDAC1 abundance in men with HIV (n = 30).

Characteristic	Grip Strength (kg)	Waist circumference (cm)	MET score
Fibrosis	0.76	0.95	0.32
Type I (%)	0.66	0.18	0.36
Type IIa (%)	0.92	0.21	0.49
Type IIx (%)	0.43	0.39	0.39
IMCL	0.87	0.89	0.41
$Pax7^+$	0.18	0.26	0.13

Supplementary Table 4. Associations between skeletal muscle factors and body function parameters.
Spearman’s unadjusted univariant correlation analysis with continuous physical performance variables and skeletal muscle factors in men with HIV (n = 30).

Characteristic	Log10(CI deficiency)		Log10(CIV deficiency)		VDAC1 abundance		Type I (%)		Type IIa (%)		Type IIx (%)		Pax7		Fibrosis		Regeneration		Degeneration		IMCL		Lipofuscin frequency	
	r	p	r	p	r	p	r	p	r	p	r	p	r	p	r	P	r	p	r	p	r	p	r	p
Log10(CI deficiency)	-	-	0.57	<0.001	0.25	0.18	-0.30	0.11	0.13	0.48	0.38	0.04	0.22	0.25	0.068	0.72	0.37	0.043	0.24	0.21	0.015	0.94	0.25	0.19
Log10(CIV deficiency)			-	-	0.15	0.43	-0.16	0.39	0.004	0.98	0.28	0.14	0.49	0.0056	0.32	0.069	0.47	0.008	0.32	0.085	0.18	0.33	0.38	0.041
VDAC1 abundance					-	-	-0.012	0.95	-0.12	0.53	0.072	0.71	-0.006	0.98	-0.038	0.84	-0.025	0.90	-0.11	0.55	-0.055	0.77	-0.12	0.54
Type I (%)							-	-	-0.85	<0.001	-0.75	<0.001	-0.15	0.44	-0.045	0.81	-0.12	0.54	0.26	0.17	-0.13	0.49	-0.01	0.76
Type IIa (%)									-	-	0.35	0.056	0.016	0.93	0.005	0.98	-0.016	0.94	-0.25	0.18	-0.24	0.21	0.012	0.95
Type IIx (%)											-	-	0.29	0.13	0.059	0.76	0.30	0.11	-0.095	0.62	0.44	0.02	0.072	0.71
Pax7													-	-	0.53	0.002	0.54	0.002	0.11	0.58	0.015	0.94	0.095	0.63
Fibrosis															-	-	0.55	0.002	0.28	0.14	-0.064	0.74	-0.053	0.79
Regeneration																	-	-	0.25	0.19	-0.017	0.93	-0.048	0.81
Degeneration																			-	-	-0.15	0.44	0.082	0.67
IMCL																					-	-	0.10	0.61
Lipofuscin frequency																							-	-

Supplementary Table 5. Associations between cellular skeletal muscle pathology in men with HIV.
Spearman's unadjusted univariant correlation analysis with cellular skeletal muscle pathological factors in men with HIV (n = 30).

Characteristic	IL-15		IL-10		IL-8		IL-6		TNF-α		FGF21		FGF23		IP-10		Leptin		MCP-4	
	r	p	r	p	r	p	r	p	r	p	r	p	r	p	r	p	r	p	r	p
Age	-0.254	0.18	-0.22	0.24	-0.15	0.43	-0.34	0.06	-0.24	0.21	-0.11	0.56	-0.20	0.29	-0.25	0.19	-0.28	0.13	-0.047	0.80
Months diagnosis	0.11	0.55	0.09	0.64	-0.08	0.68	0.19	0.31	0.09	0.65	0.21	0.26	-0.07	0.72	-0.01	0.96	-0.29	0.12	0.13	0.48
Months ART	-0.048	0.8	-0.15	0.44	-0.09	0.64	-0.001	0.99	0.16	0.41	-0.006	0.98	-0.39	0.035	-0.20	0.30	-0.16	0.39	0.23	0.22
CD4 (cells/µl)	-0.24	0.91	0.21	0.28	0.27	0.17	0.12	0.53	0.24	0.21	0.41	0.032	0.018	0.93	0.26	0.18	0.19	0.33	0.24	0.19
# Comorbid	-0.27	0.15	-0.04	0.84	0.26	0.16	-0.01	0.96	0.15	0.45	0.024	0.90	0.20	0.28	0.087	0.65	0.021	0.91	0.17	0.38
# Medications	0.23	0.22	0.13	0.50	0.16	0.39	0.094	0.62	0.04	0.84	0.006	0.98	0.21	0.27	0.015	0.94	0.028	0.88	0.14	0.47
BMI	-0.002	0.99	0.28	0.13	0.39	0.03	-0.024	0.90	-0.08	0.67	0.34	0.064	0.21	0.26	0.15	0.42	0.61	<0.0001	-0.036	0.85
MET score	0.23	0.23	-0.005	0.98	-0.13	0.50	-0.03	0.87	-0.23	0.23	-0.006	0.98	-0.17	0.37	-0.081	0.67	-0.12	0.52	-0.28	0.13
SPPB score	-0.15	0.44	0.14	0.46	0.089	0.64	-0.28	0.13	-0.28	0.14	-0.31	0.094	0.012	0.95	-0.17	0.38	-0.27	0.15	-0.24	0.20
FFP score	-0.021	0.91	0.18	0.35	0.22	0.24	0.19	0.31	0.08	0.67	0.11	0.57	0.17	0.38	0.13	0.48	-0.14	0.47	0.058	0.76
Grip strength	-0.011	0.95	-0.28	0.14	-0.14	0.47	-0.26	0.16	0.016	0.93	-0.13	0.50	-0.02	0.91	-0.21	0.27	0.016	0.93	-0.047	0.81
AMSI	-0.035	0.86	0.29	0.13	0.20	0.29	-0.31	0.09	-0.06	0.74	-0.17	0.38	0.066	0.73	-0.11	0.55	0.024	0.90	-0.028	0.88
% Fat	0.043	0.82	0.012	0.95	0.003	0.99	0.15	0.44	0.24	0.2	0.32	0.08	0.061	0.75	0.021	0.91	0.80	<0.0001	0.10	0.59
% Lean	-0.043	0.82	-0.012	0.95	-0.003	0.99	-0.15	0.44	-0.24	0.2	-0.32	0.08	-0.061	0.75	-0.021	0.91	-0.80	<0.0001	-0.10	0.59

Supplementary Table 6. Clinical predictors of cytokine and myokine levels. Spearman’s unadjusted univariant correlation analysis was performed to determine whether levels of the various inflammatory cytokines or myokines predicted clinical parameters in men with HIV.

Characteristic	IL-15		IL-10		IL-8		IL-6		TNF-α		FGF21		FGF23		IP-10		Leptin		MCP-4	
	r	p	r	p	r	p	r	p	r	p	r	p	r	p	r	p	r	p	r	p
Log10(CI deficiency)	0.002	0.99	-0.065	0.73	0.18	0.34	0.10	0.6	0.068	0.72	0.026	0.89	0.046	0.81	0.12	0.54	-0.18	0.34	-0.039	0.84
Log10(CIV deficiency)	-0.06	0.75	-0.004	0.99	0.12	0.54	-0.012	0.95	0.016	0.93	-0.10	0.60	0.011	0.95	0.059	0.76	-0.23	0.21	0.15	0.44
VDAC1 abundance	0.12	0.53	0.25	0.19	0.34	0.069	-0.21	0.26	-0.17	0.38	-0.13	0.48	-0.14	0.46	-0.013	0.95	-0.32	0.086	-0.11	0.57
Fibre area	-0.18	0.35	-0.24	0.20	-0.27	0.15	-0.13	0.49	-0.19	0.29	-0.21	0.26	-0.06	0.76	-0.25	0.19	-0.006	0.98	-0.15	0.43
% Type I	-0.046	0.81	-0.21	0.28	-0.37	0.046	-0.29	0.12	-0.36	0.052	-0.31	0.10	-0.39	0.036	-0.36	0.052	-0.12	0.52	-0.06	0.75
% Type IIa	0.15	0.43	0.22	0.24	0.15	0.42	0.19	0.33	0.33	0.08	0.16	0.41	0.21	0.27	0.21	0.27	0.19	0.31	0.014	0.94
% Type IIx	0.075	0.70	0.21	0.27	0.48	0.007	0.37	0.044	0.25	0.18	0.42	0.019	0.45	0.013	0.37	0.045	0.11	0.55	0.21	0.27
IMCL	-0.36	0.049	-0.027	0.89	0.37	0.045	0.16	0.39	0.13	0.48	0.21	0.26	0.31	0.093	0.31	0.10	0.018	0.92	0.23	0.22
Lipo area	-0.17	0.37	0.14	0.48	0.19	0.32	-0.096	0.62	-0.06	0.77	0.31	0.098	-0.043	0.82	0.009	0.96	0.011	0.95	-0.12	0.54
Pax7	0.055	0.78	-0.19	0.31	-0.016	0.93	-0.039	0.84	0.15	0.44	0.035	0.85	-0.026	0.89	-0.06	0.75	0.072	0.70	0.15	0.42
Fibrosis	-0.005	0.98	-0.069	0.72	-0.011	0.95	-0.097	0.61	-0.20	0.28	-0.27	0.16	0.055	0.77	0.07	0.72	-0.23	0.22	-0.10	0.61
Regen	0.13	0.48	0.027	0.89	0.14	0.47	0.067	0.73	0.031	0.87	0.087	0.65	0.20	0.28	0.12	0.51	-0.028	0.89	-0.045	0.81
Degen	0.019	0.92	-0.19	0.32	-0.35	0.058	-0.23	0.23	-0.06	0.77	-0.25	0.19	-0.057	0.76	-0.27	0.13	-0.14	0.46	-0.03	0.87

Supplementary Table 7. Pathophysiological skeletal muscle predictors of cytokine and myokine levels.
Spearman’s unadjusted univariant correlation analysis was performed to determine whether levels of the various inflammatory cytokines or myokines predicted skeletal muscle function factors in men with HIV.
